# Supplementary material for: Dynamics of the fcc-to-bcc phase transition in single-crystalline PdCu alloy nanoparticles
Source: Nat Commun. 2023 Jan 6;14:104. doi: 10.1038/s41467-022-35325-y (PMC9822937; doi:10.1038/s41467-022-35325-y)
Supplement: Supplementary file 1 — Supplementary Information [file 41467_2022_35325_MOESM1_ESM.pdf]

Supplementary Information for

Dynamics of the fcc-to-bcc phase transition in single-crystalline PdCu alloy nanoparticles

Yingying Jiang[1,2], Martial Duchamp[3], Shi Jun Ang[4], Hongwei Yan[1,2], Teck Leong Tan[4], and Utkur Mirsaidov[1,2,5,6]*

1. Department of Physics, National University of Singapore, Singapore 117551, Singapore
2. Centre for BioImaging Sciences, Department of Biological Sciences, National University of Singapore, Singapore 117557, Singapore
3. School of Materials Science and Engineering, Nanyang Technological University, Singapore 639798, Singapore
4. Institute of High Performance Computing, Agency for Science, Technology and Research, Singapore 138632, Singapore
5. Centre for Advanced 2D Materials and Graphene Research Centre, National University of Singapore, Singapore 117546, Singapore
6. Department of Materials Science and Engineering, National University of Singapore, Singapore 117575, Singapore

*Correspondence: mirsaidov@nus.edu.sg

Supplementary Figures

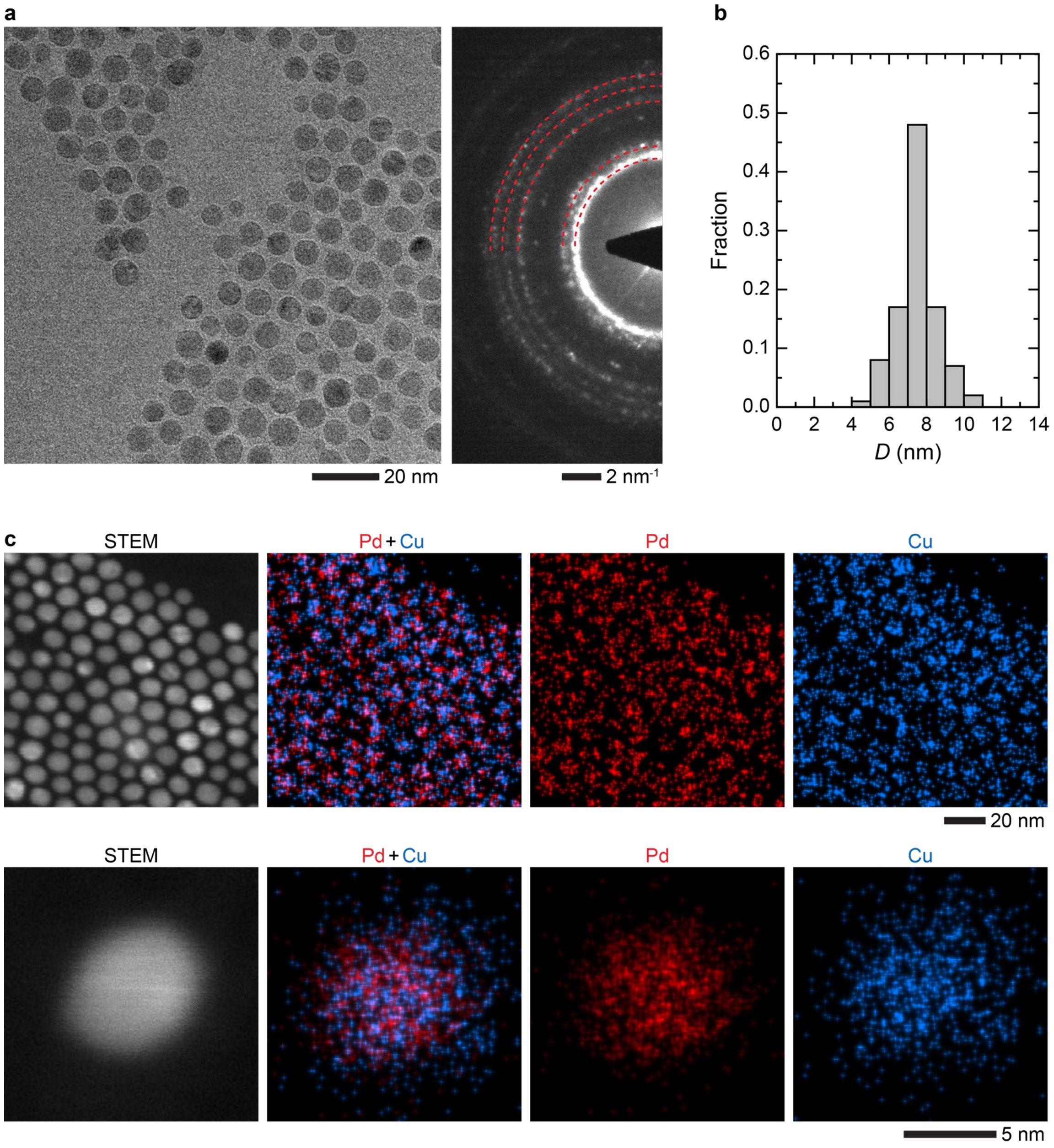

Supplementary Figure 1. TEM, diffraction, STEM, and EDX images of as-synthesized PdCu alloy NPs. (**a**) TEM and diffraction images of the NPs. Diffraction pattern of the NPs showing five diffraction rings (dashed red quarter circles) at 2.2, 1.9, 1.3, 1.2, and 1.1 Å corresponding to {111}, {200}, {220}, {311}, and {222} lattice planes of an fcc PdCu alloy, respectively (Supplementary Table 1). (**b**) The distribution of NP diameters showing that their average diameter is 8 ± 1 nm. (**c**) STEM images and corresponding EDX maps of the PdCu NPs. The top and bottom panels show the images of an ensemble of the NPs and of an individual NP, respectively. The NPs comprise 46% (at.) Pd and 54% (at.) Cu.

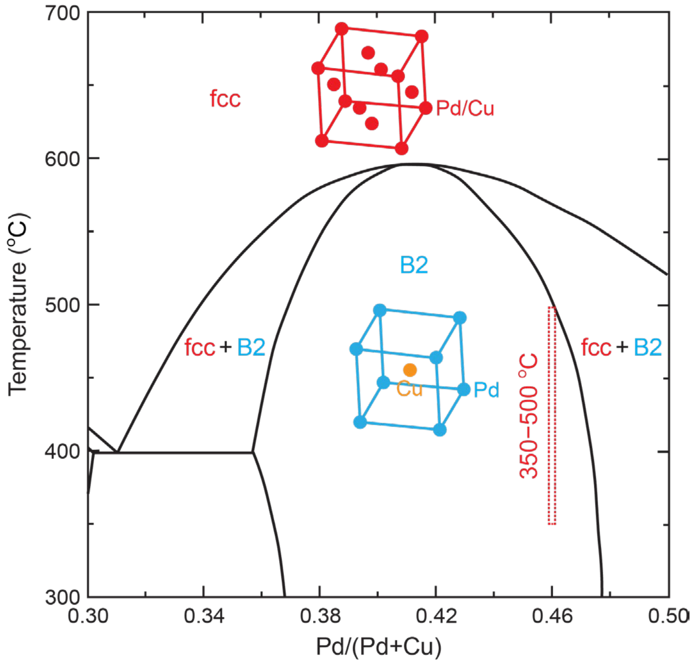

Supplementary Figure 2. Phase diagram of PdCu alloy system. The phase diagram adapted from ref. 1. At the atomic composition of 46% Pd and 54% Cu, the stable phase at 350−500 °C (dashed red rectangle) is the B2 phase.

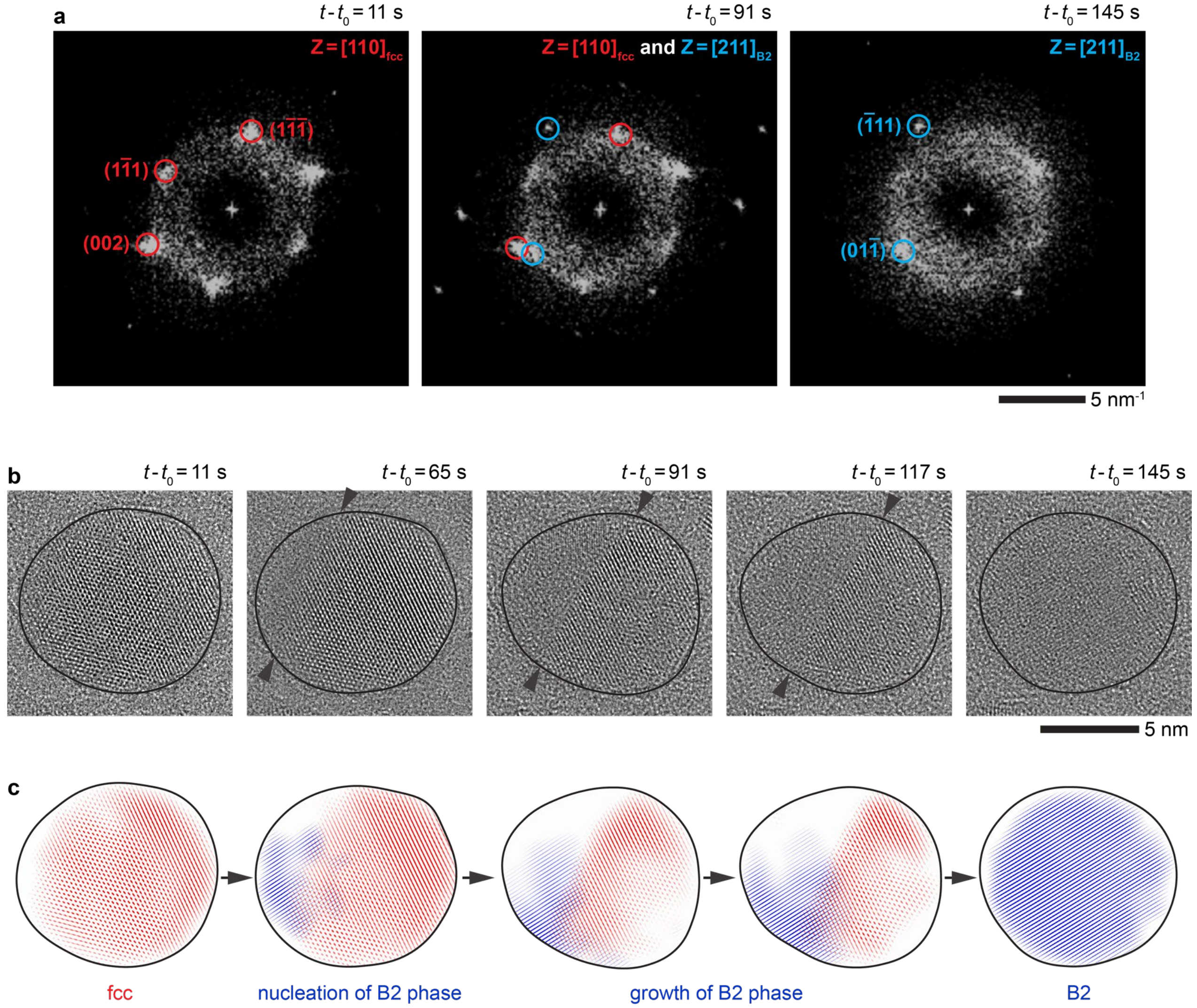

Supplementary Figure 3. Phase transition of an fcc PdCu alloy NP into a B2 NP. (**a**) FFT and (**b**) TEM image series of an fcc PdCu NP transforming into a B2 NP at 500 °C when viewed from $[110]_{fcc}$ and $[211]_{B2}$ zone axes (Supplementary Movie 2). The black arrows in (b) indicate the locations of the moving fcc−B2 interface during the phase transition. (**c**) Sequence of inverse FFT images corresponding to (b) showing the fcc (red) and B2 (blue) regions during the NP transformation. t_0 is the timepoint at which we started recording the process.

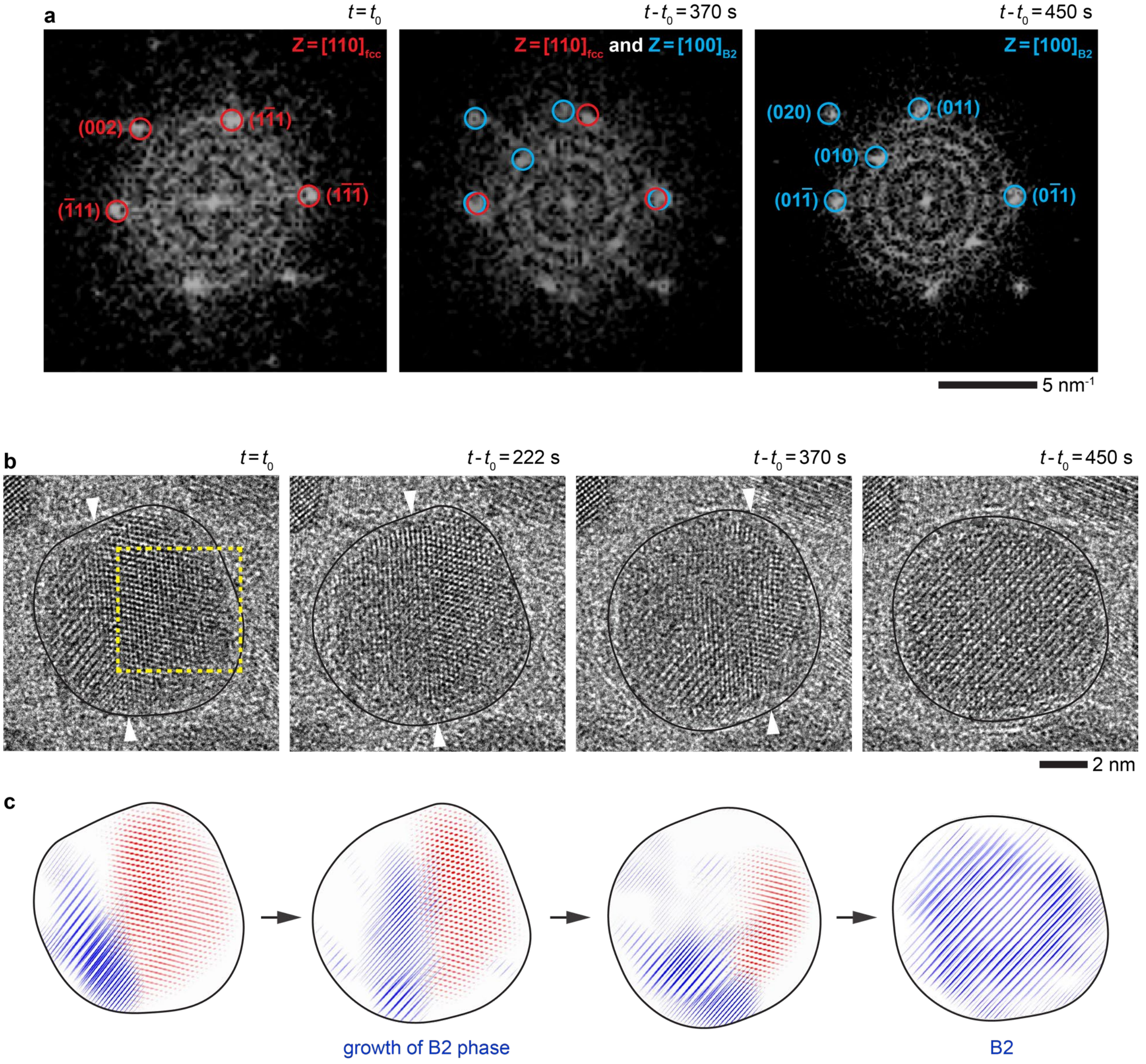

Supplementary Figure 4. Phase transition of an fcc PdCu alloy NP into a B2 NP. (**a**) FFT (from the dashed yellow box) and (**b**) TEM image series of an fcc PdCu NP transforming into a B2 NP at 500 °C viewed from $[110]_{fcc}$ and $[100]_{B2}$ zone axes (Supplementary Movie 3). The white arrows in (b) indicate the locations of the moving fcc−B2 interface during the heating. (**c**) Sequence of inverse FFT images corresponding to the TEM image series showing the fcc (red) and B2 (blue) regions during the NP transformation. t_0 is the timepoint at which we started recording the process.

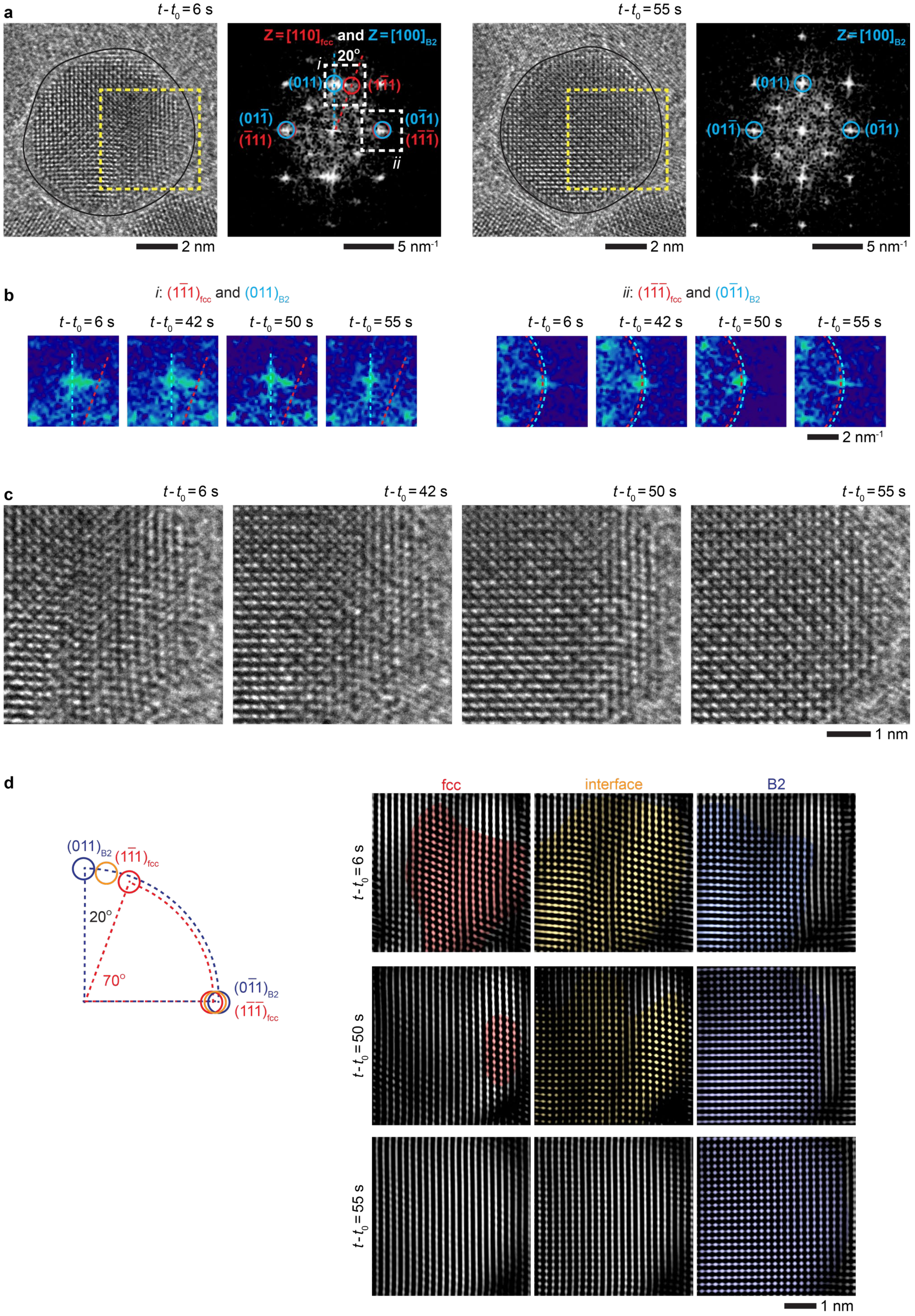
a
t-t0=6 s
Z=[110]fcc and Z=[100]B2
20°
i
(011)
(1$\bar{1}$1)
(01$\bar{1}$)
($\bar{1}$11)
(0$\bar{1}$1)
(1$\bar{1}\bar{1}$)
ii
2 nm
5 nm-1
t-t0=55 s
Z=[100]B2
(011)
(01$\bar{1}$)
(0$\bar{1}$1)
2 nm
5 nm-1
b
i: (1$\bar{1}$1)fcc and (011)B2
ii: (1$\bar{1}\bar{1}$)fcc and (0$\bar{1}$1)B2
t-t0=6 s
t-t0=42 s
t-t0=50 s
t-t0=55 s
2 nm-1
c
t-t0=6 s
t-t0=42 s
t-t0=50 s
t-t0=55 s
1 nm
d
(011)B2
(1$\bar{1}$1)fcc
20°
70°
(0$\bar{1}$1)B2
(1$\bar{1}\bar{1}$)fcc
fcc
interface
B2
t-t0=6 s
t-t0=50 s
t-t0=55 s
1 nm

Supplementary Figure 5. Structure and dynamics of fcc−B2 interface. (**a**) TEM and FFT images (from the dashed yellow boxes), when viewed from $[110]_{\mathrm{fcc}}$ and $[100]_{\mathrm{B2}}$ zone axes, showing the evolution of the interface in the NP (t - t_0 = 6 s) as it transforms into a B2 PdCu alloy NP (t - t_0 = 55 s) at 500 °C. (**b**) The two enlarged views marked in (a) (dashed white boxes) showing fcc and B2 spots during the phase transition ($(1\bar{1}1)_{\mathrm{fcc}}$−$(011)_{\mathrm{B2}}$ spots are connected by the "trace," while $(1\bar{1}\bar{1})_{\mathrm{fcc}}$ and $(0\bar{1}1)_{\mathrm{B2}}$ spots overlap). The dashed red and blue lines correspond to the directions of $(1\bar{1}1)_{\mathrm{fcc}}$ and $(011)_{\mathrm{B2}}$ planes, respectively, and the angle between these two planes is 20°. The dashed red and blue arcs correspond to the reciprocals of fcc and B2 lattice spacings, respectively (*i.e.*, $k_{\{111\}_{\mathrm{fcc}}}$ = 4.6 nm^{-1}, $k_{\{110\}_{\mathrm{B2}}}$ = 4.8 nm^{-1}). (**c**) Sequence of TEM and (**d**) inverse FFT images from the dashed yellow boxes in (a) showing the movement of the fcc−B2 interface (Supplementary Movie 5). The fcc, B2, and interface regions in (d) are false-colored in red, blue, and orange, respectively. In the left schematic illustration, the red, orange, and blue circles represent the selected areas of fcc, interface, and B2 FFT peaks that serve as the masks for the inverse FFT images. Note that the $(1\bar{1}\bar{1})_{\mathrm{fcc}}$ and $(0\bar{1}1)_{\mathrm{B2}}$ spots overlap, resulting in the shared vertical fringes of the fcc, interface, and B2 regions in the inverse FFT images. t_0 is the timepoint at which we started recording the process.

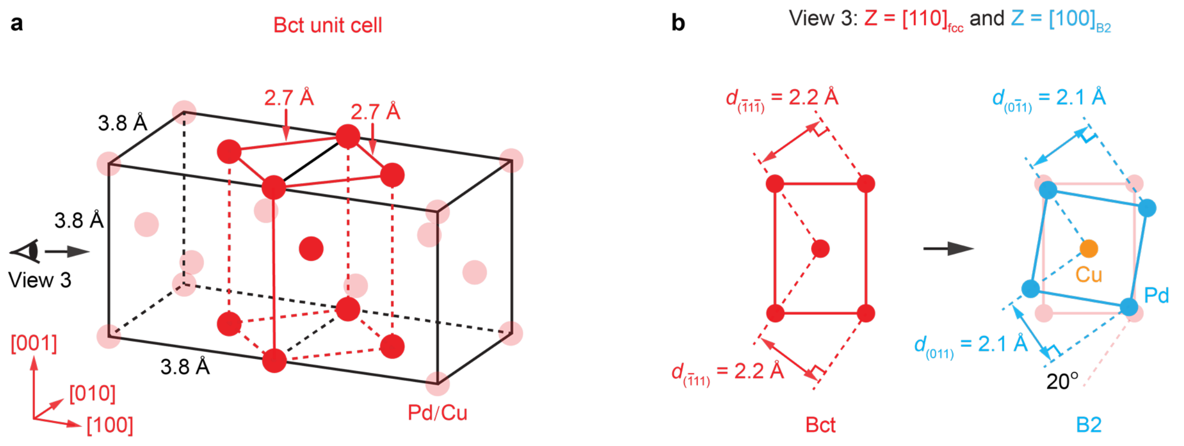

Supplementary Figure 6. Schematic illustration of fcc-to-B2 phase transition supplementing Figure 3. (**a**) The atomic configurations of a bct unit cell (red frames) constructed from two fcc unit cells (black frames). (**b**) The projected views of the unit cells when viewed from $[110]_{fcc}$ and $[100]_{B2}$ zone axes.

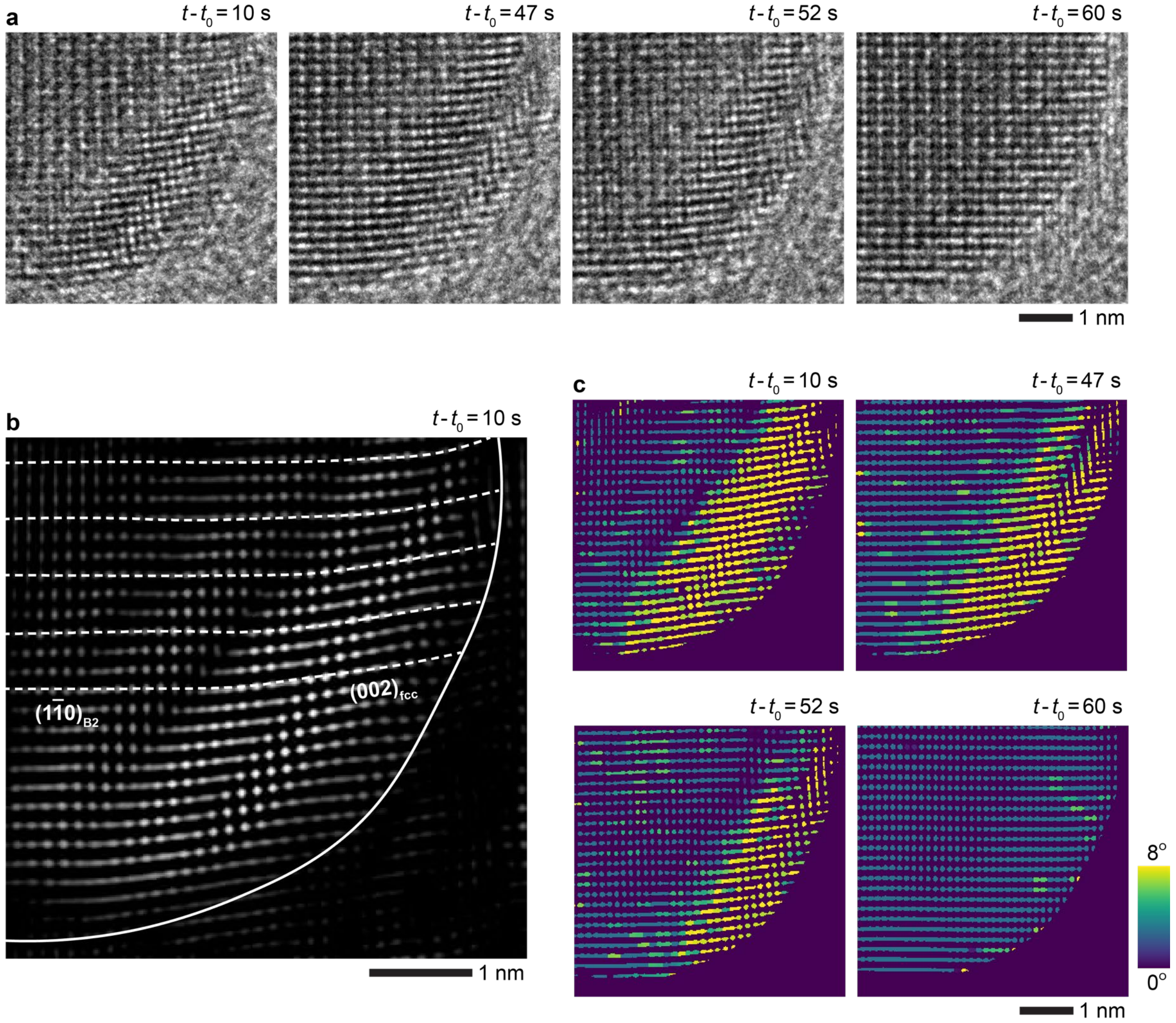

Supplementary Figure 7. Detailed view of the fcc–B2 interface of the NP shown in Figure 2c. (**a**) TEM images corresponding to the image series in Figure 2c. (**b**) Inverse FFT image showing how $(002)_{\mathrm{fcc}}$ and $(1\bar{1}0)_{\mathrm{B2}}$ are linked via an interface (dashed white lines). (**c**) Maps of atomic columns showing the bending of the lattice planes as NP fully transforms into a B2 NP. The angle map is plotted with respect to horizontal $(1\bar{1}0)_{\mathrm{B2}}$ planes (*i.e.*, the direction of $(1\bar{1}0)_{\mathrm{B2}}$ planes is set as 0°).

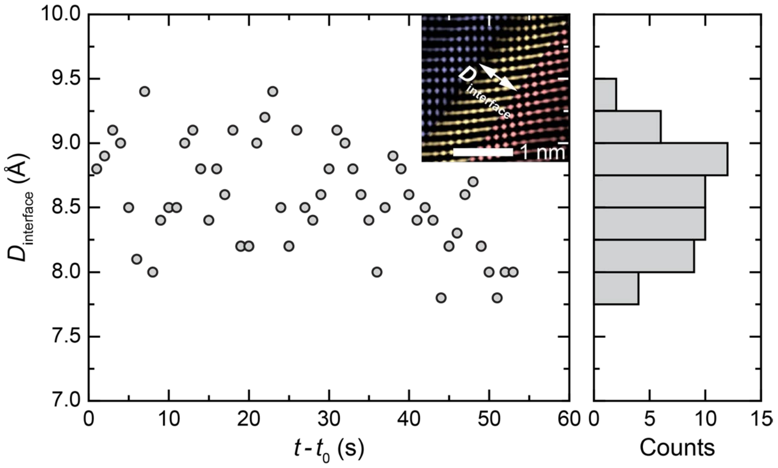

Supplementary Figure 8. Width of the fcc−B2 interface for the NP shown in Figure 2c. The width of the fcc−B2 interface for the NP shown in Figure 2c throughout the phase transition is 8.6 ± 0.4 Å.

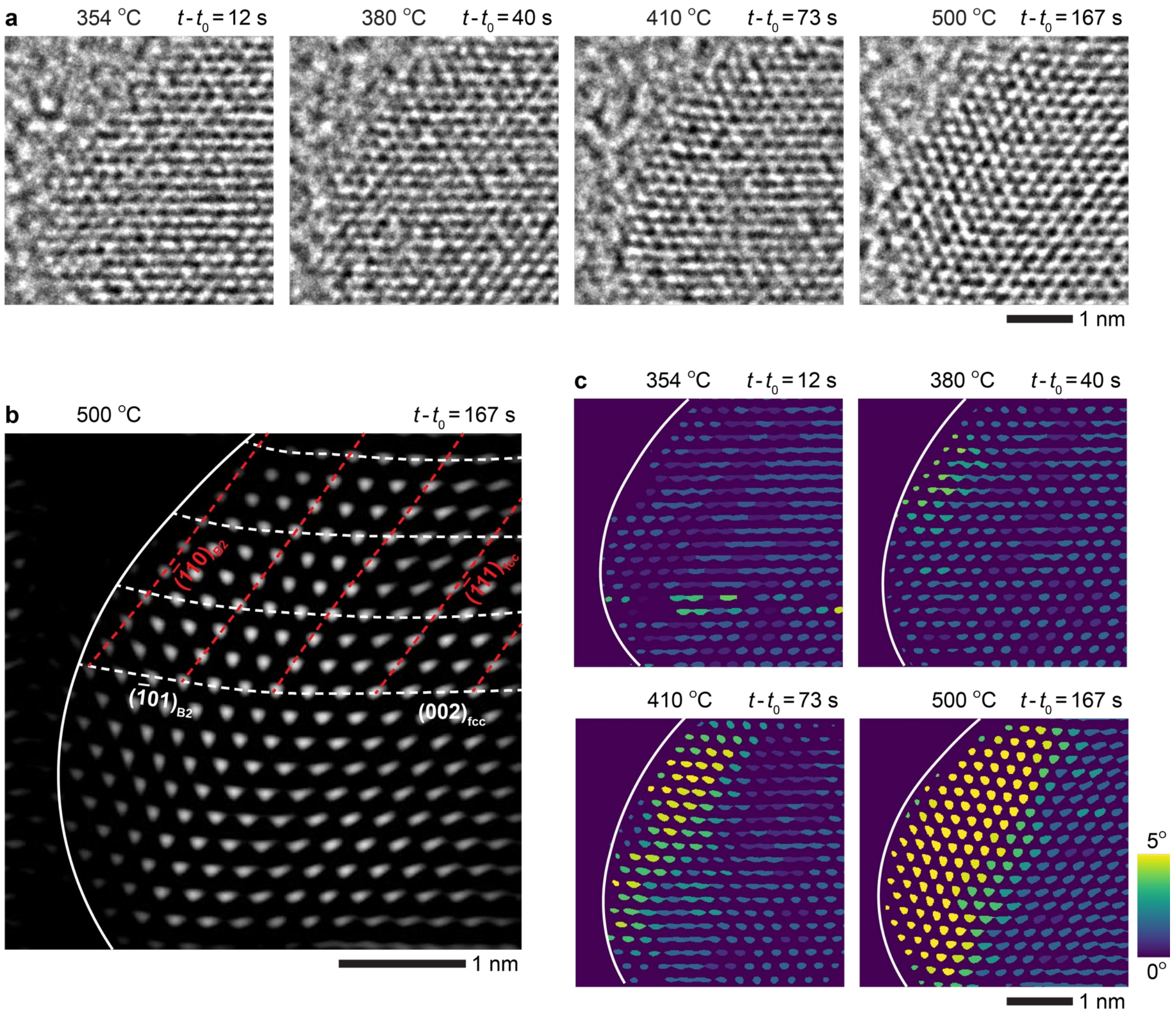

Supplementary Figure 9. Detailed view of the interface of the NP shown in Figure 5b. (**a**) TEM images corresponding to Figure 5b. (**b**) Inverse FFT image showing how $(002)_{fcc}$ and $(\bar{1}01)_{B2}$ (dashed white lines) and $(\bar{1}11)_{fcc}$ and $(\bar{1}10)_{B2}$ (dashed red lines) are linked via a coherent interface. (**c**) Maps of atomic columns showing the bending of the lattice planes as NP fully transforms into a B2 NP. The angle map is plotted with respect to horizontal $(002)_{fcc}$ planes (*i.e.*, the direction of $(002)_{fcc}$ planes is set as 0°).

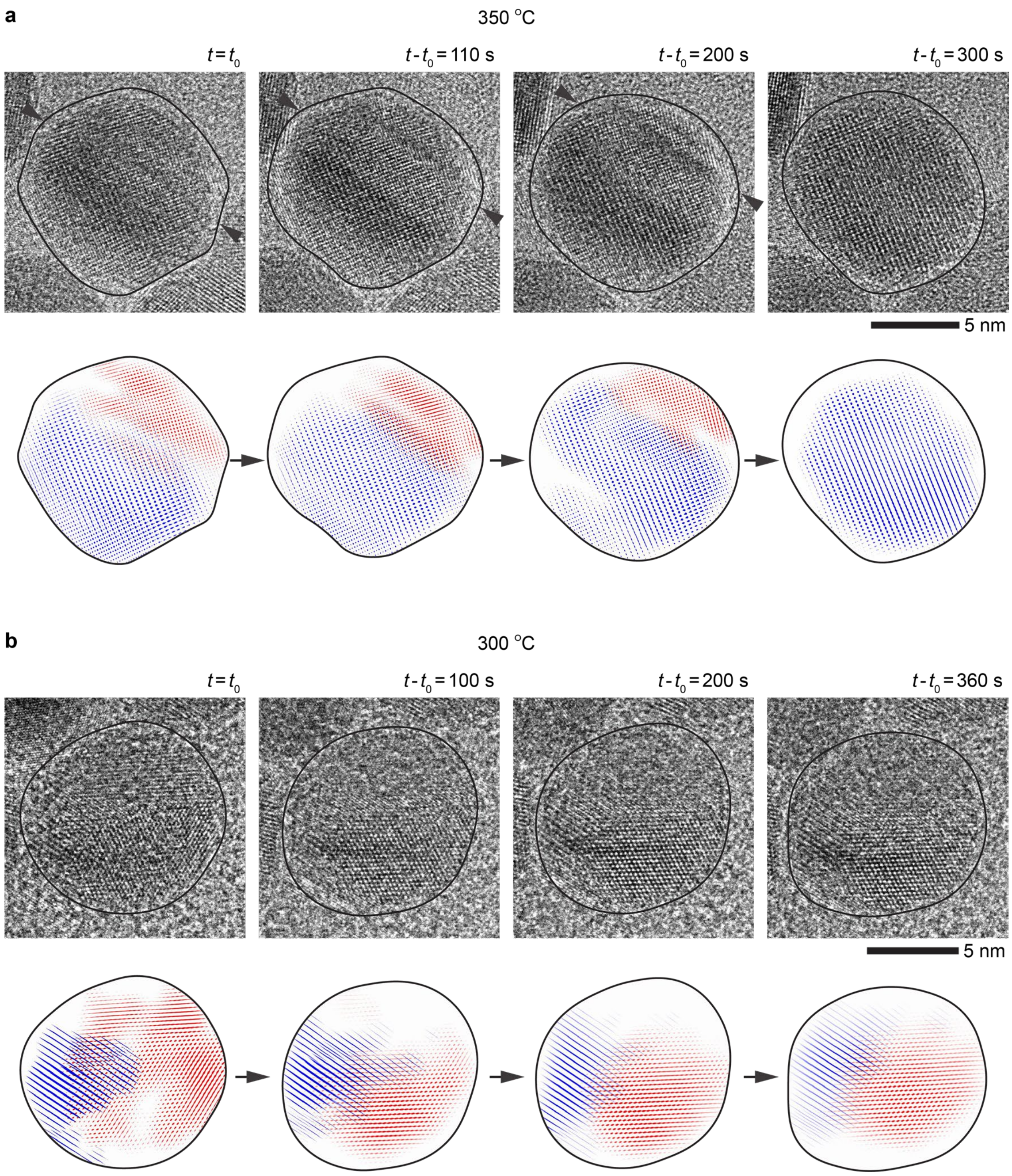

Supplementary Figure 10. Phase transition of PdCu NPs at different heating temperatures. TEM image series of two PdCu NPs at (**a**) 350 °C when viewed from $[100]_{fcc}$ and $[110]_{B2}$ zone axes and (**b**) 300 °C when viewed from $[110]_{fcc}$ and $[110]_{B2}$ zone axes, respectively. The black arrows in the TEM images in (a) indicate the locations of the moving fcc−B2 interface during the heating. (Lower panels) Sequence of inverse FFT images corresponding to the TEM image series showing the fcc (red) and B2 (blue) regions during the NP transformation. t_0 is the timepoint at which we started recording the process.

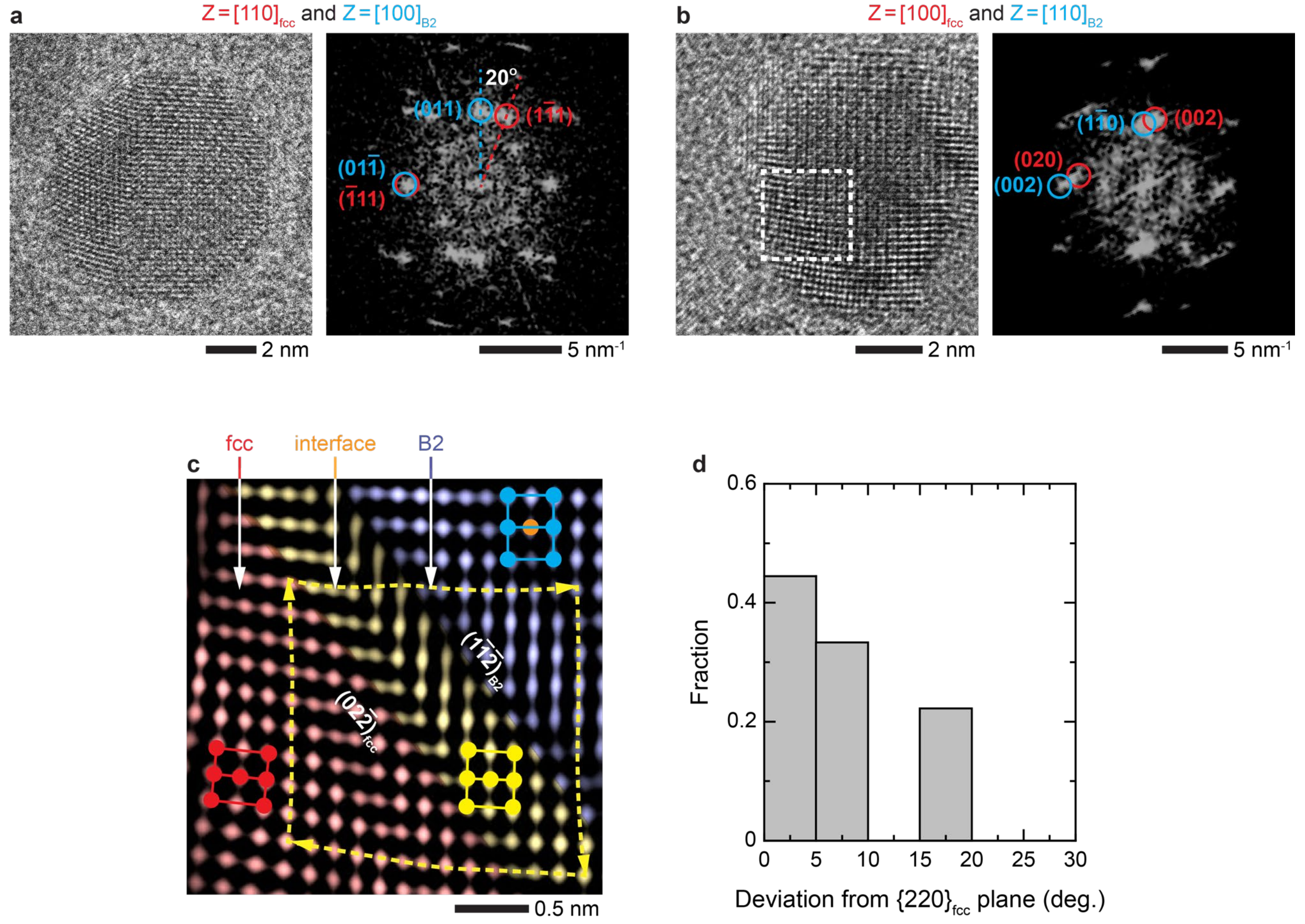

Supplementary Figure 11. TEM images of the PdCu NPs with fcc and B2 phases. The NPs (with fcc and B2 phases) were obtained by first heating the fcc NPs at 350 °C for 15 min, followed by cooling to an ambient temperature. TEM and FFT images of two such NPs when viewed from (**a**) $[110]_{\mathrm{fcc}}$ and $[100]_{\mathrm{B2}}$ and (**b**) $[100]_{\mathrm{fcc}}$ and $[110]_{\mathrm{B2}}$ zone axes. (**c**) Inverse FFT image corresponding to the area selected by a dashed white box in (b) shows a coherent fcc−B2 interface. The closed Burgers circuit (dashed yellow lines with arrows) with the dimension of 14 atoms × 10 atoms indicates that there are no dislocations inside this region. Here, the $(02\bar{2})_{\mathrm{fcc}}$ (red region) and $(1\bar{1}\bar{2})_{\mathrm{B2}}$ (blue region) planes are connected via the coherent interface (orange region). Bct (red frame), interface (yellow frame), and B2 (blue frame) unit cells are drawn to represent the deformation of the lattice during the phase transition. (**d**) Distribution showing the extent of the alignment between the interface and $\{220\}_{\mathrm{fcc}}$ planes obtained from nine NPs viewed from $[100]_{\mathrm{fcc}}$ and $[110]_{\mathrm{B2}}$ zone axes.

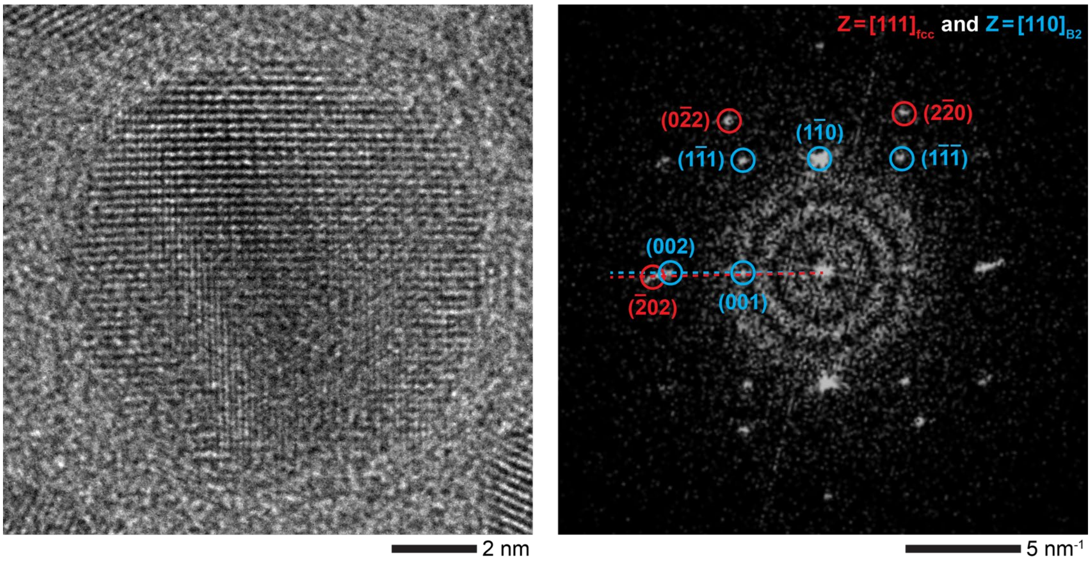

Supplementary Figure 12. Post-heating TEM image of a PdCu alloy NP with fcc and B2 phases when viewed from $[111]_{fcc}$ and $[110]_{B2}$ zone axes. TEM image and corresponding FFT pattern of the NP after heating at 350 ℃ for 15 min and cooling down to an ambient temperature. This NP was not exposed to the electron beam during the heating process and was imaged once only after the cool-down. The direction of the $(\bar{2}02)_{fcc}$ planes is the same as the direction of $(002)_{B2}$ planes.

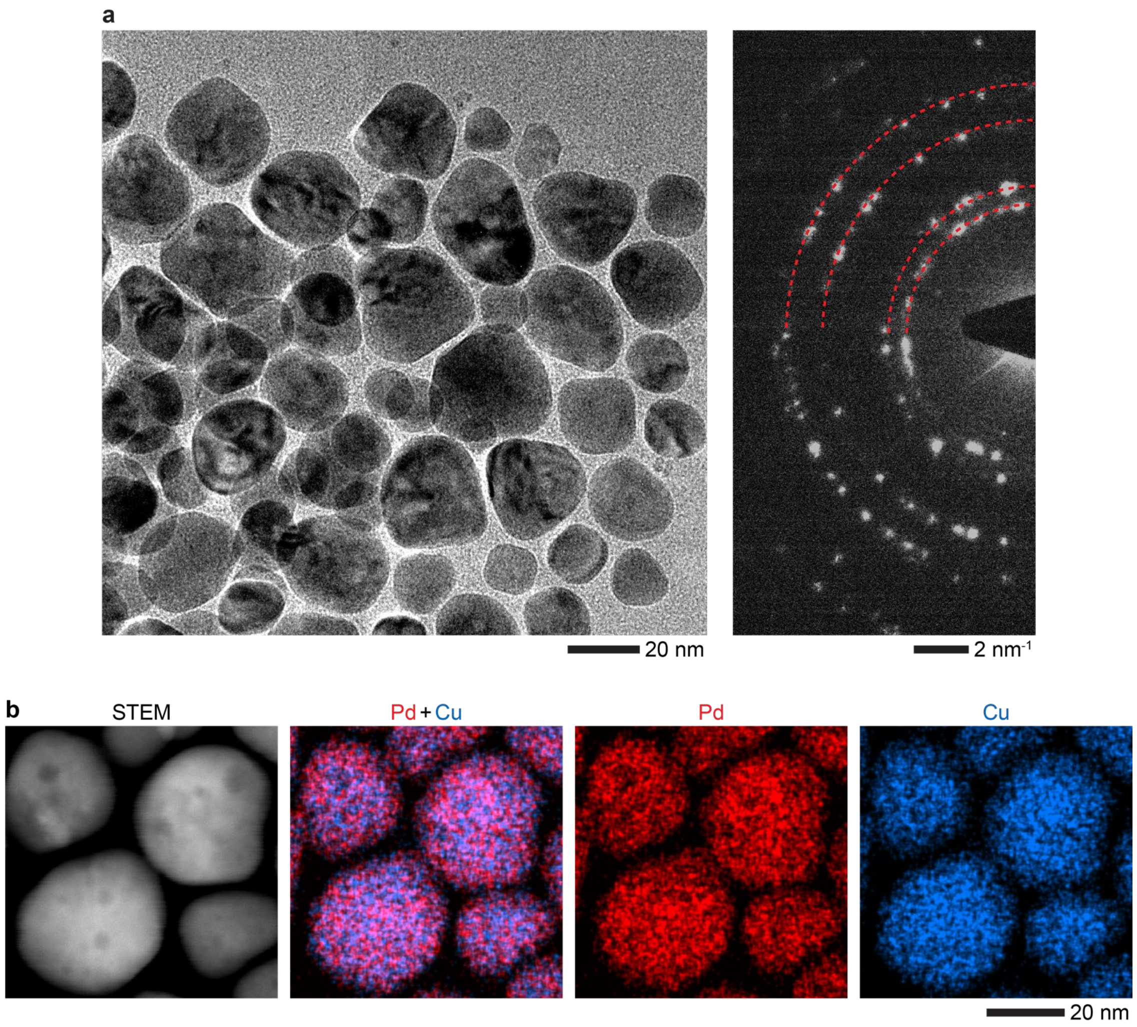

Supplementary Figure 13. TEM, diffraction, STEM, and EDX images of larger as-synthesized PdCu alloy NPs. (**a**) TEM and diffraction images of the NPs. Diffraction pattern of the NPs showing the diffraction rings (dashed red curves) at 2.2, 1.9, 1.3, and 1.1 Å corresponding to {111}, {200}, {220}, and {222} lattice planes of fcc PdCu alloy, respectively (Supplementary Table 1). (**b**) STEM image and corresponding EDX maps of the PdCu NPs. The NPs comprise 46% (at.) Pd and 54% (at.) Cu.

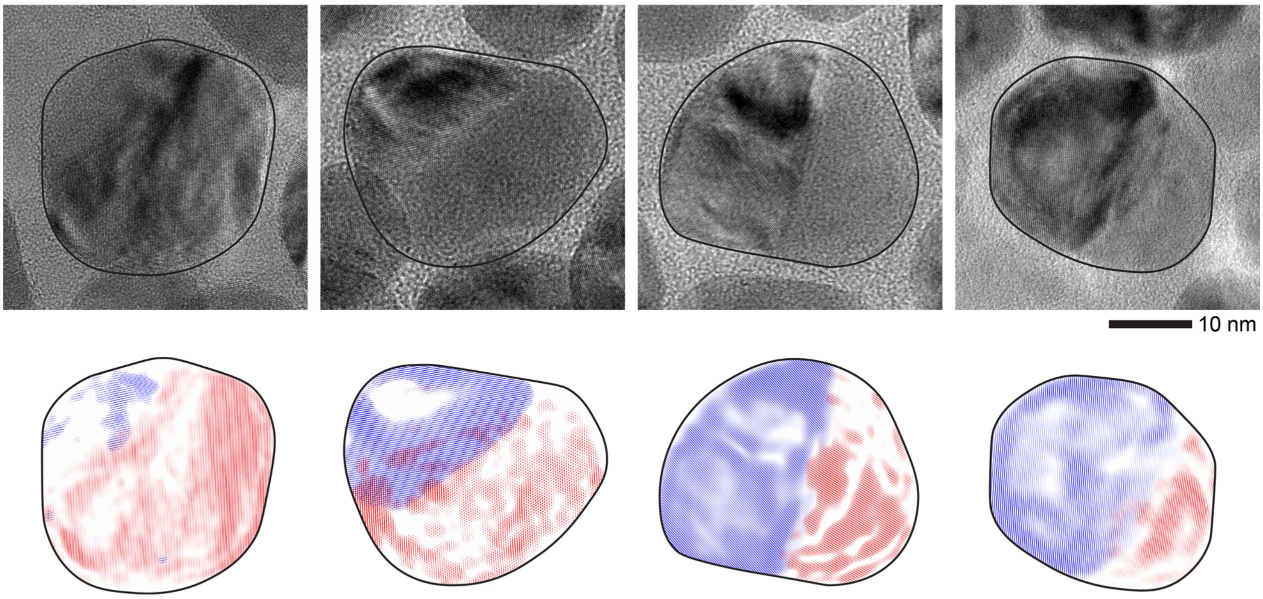

Supplementary Figure 14. Phase transition of larger PdCu NPs. (Top) Sequence of TEM and (bottom) inverse FFT images of four different NPs with a mixture of fcc (red) and B2 (blue) phases. The sizes of these NPs are larger than 30 nm. These NPs were formed by first heating the fcc NP at 350 °C for 30 min, followed by cooling to an ambient temperature.

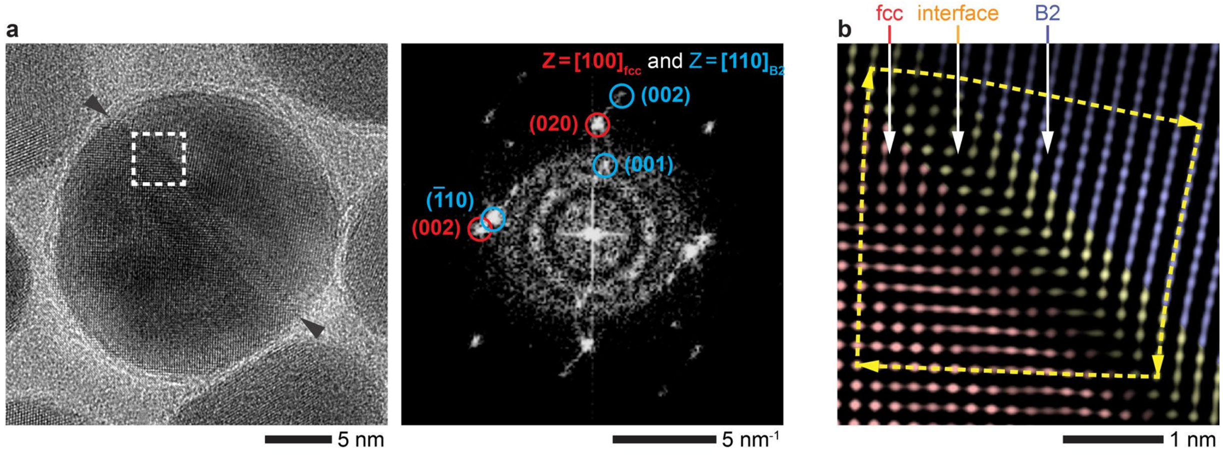

Supplementary Figure 15. TEM images of the larger PdCu NP with fcc and B2 phases. (**a**) TEM and FFT images of a 22-nm NP with fcc and B2 phases when viewed from $[100]_{fcc}$ and $[110]_{B2}$ zone axes. The NP was formed by first heating the fcc NP at 350 °C for 30 min, followed by cooling to an ambient temperature. (**b**) Inverse FFT image corresponding to the area selected by a dashed white box in (a) shows a coherent fcc−B2 interface. The closed Burgers circuit (dashed yellow lines with arrows) with the dimension of 16 atoms×16 atoms indicates that there are no dislocations inside this region.

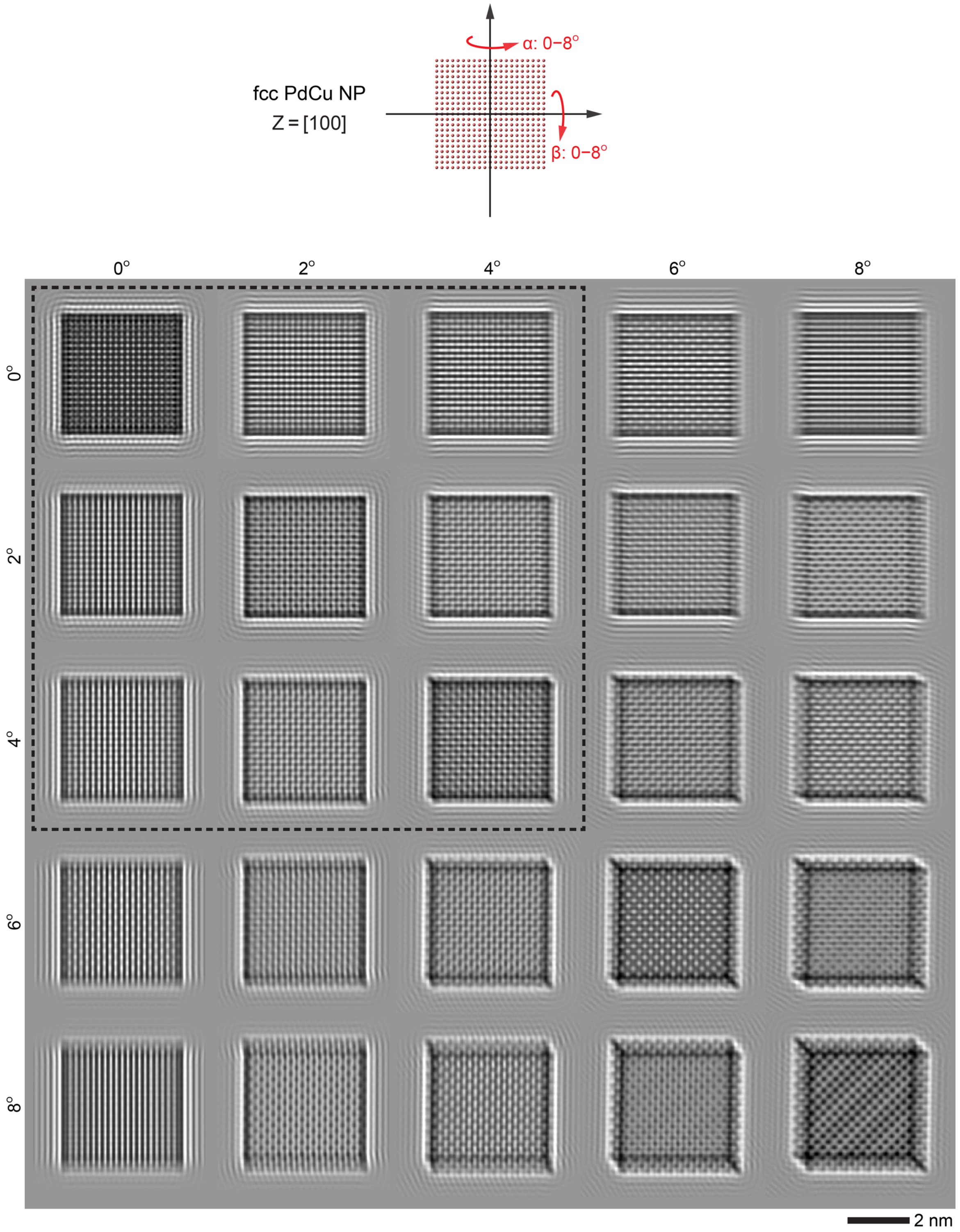

Supplementary Figure 16. Simulated TEM images of an fcc nanocrystal when viewed from different off-axis tilting conditions. The fcc nanocrystal is viewed from [100] zone axis, and its horizontal and vertical lattice fringes corresponding to (002) and (020) planes are visible in the selected area (dashed black box).

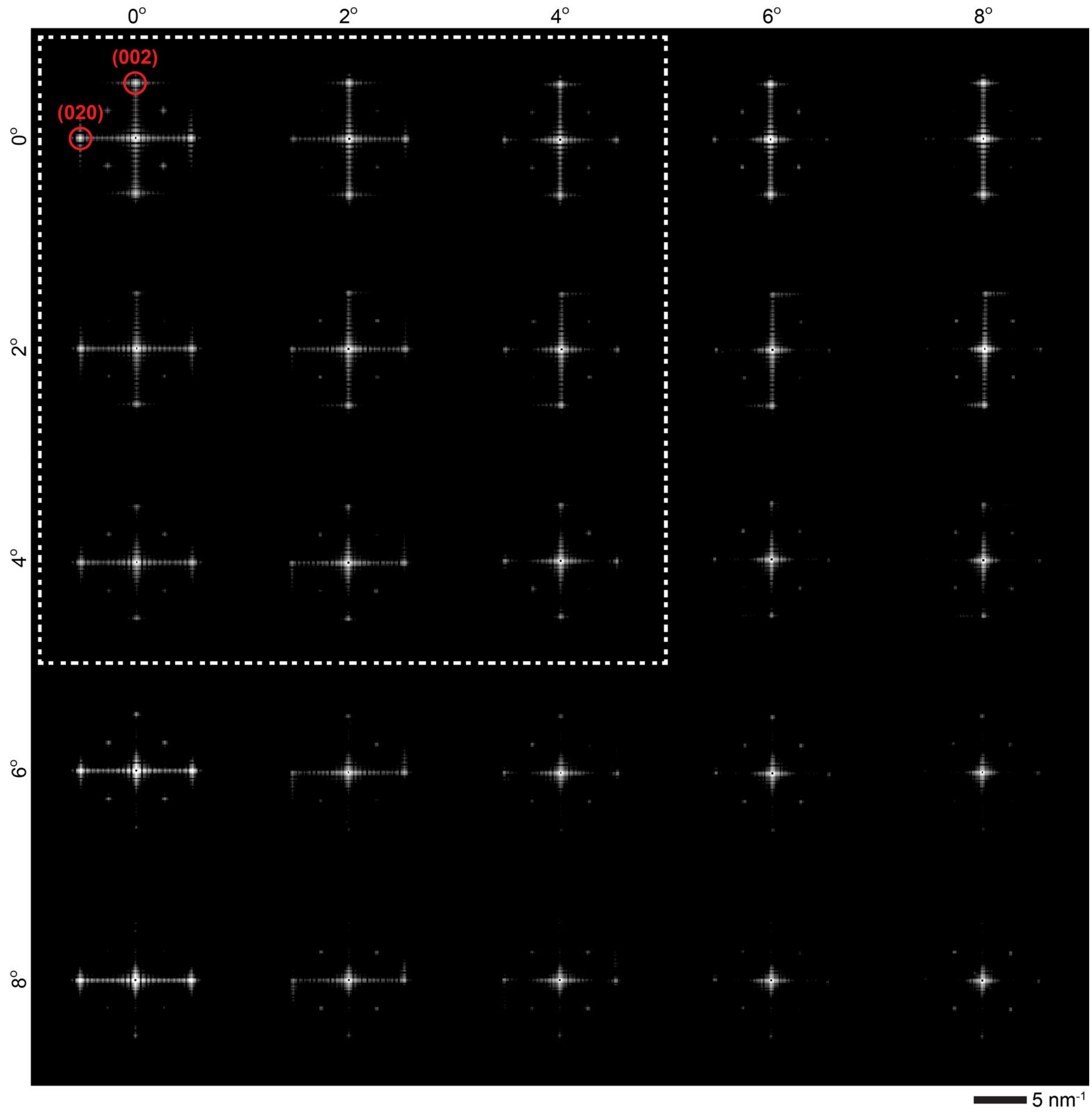

Supplementary Figure 17. FFT patterns corresponding to simulated images in Supplementary Figure 16. The (002) and (020) spots are visible in the selected area (dashed white box).

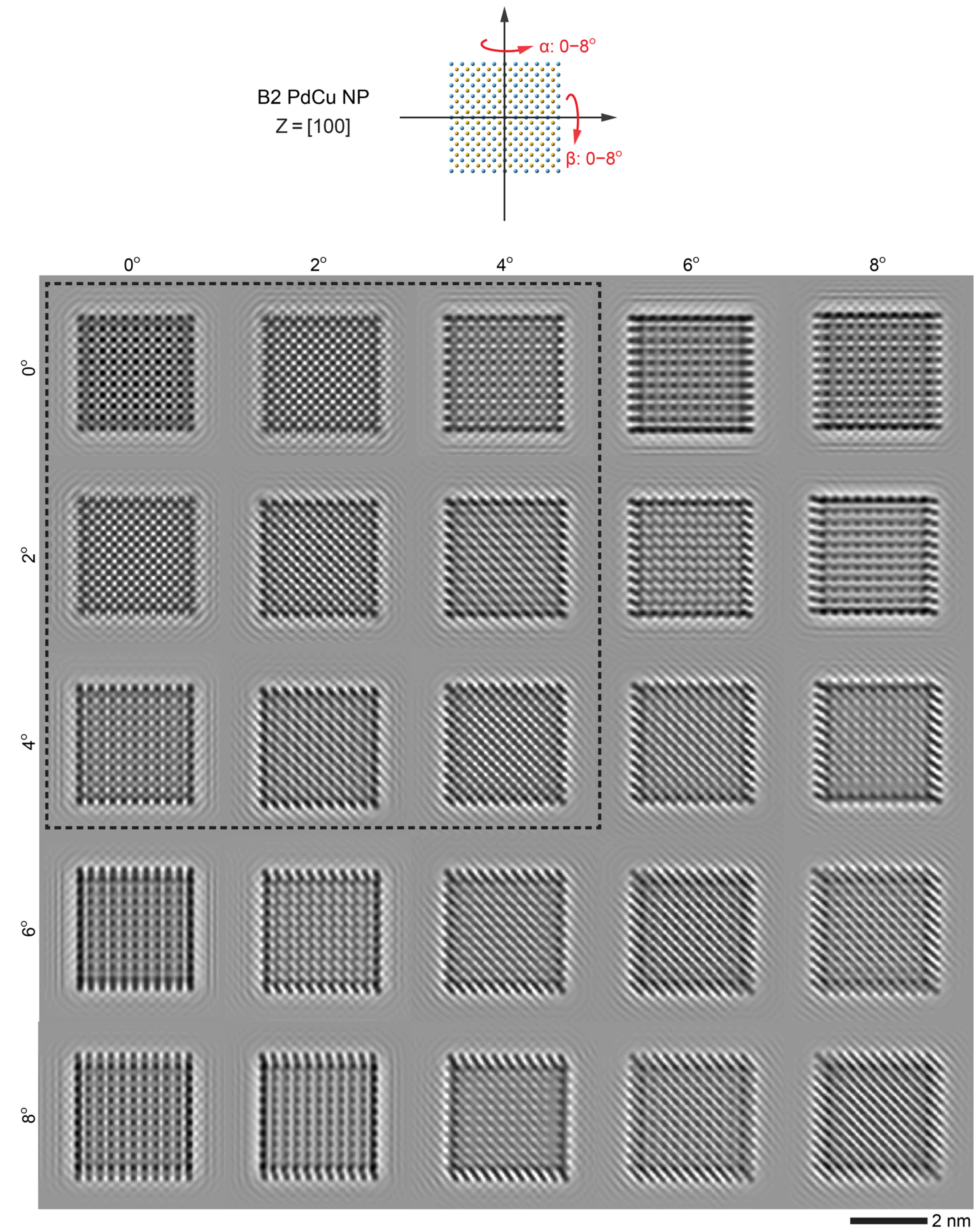

Supplementary Figure 18. Simulated TEM images of a B2 nanocrystal when viewed from different off-axis tilting conditions. The B2 nanocrystal is viewed from [100] zone axis, and its two diagonal lattice fringes corresponding to (011) and $(0\bar{1}1)$ planes are visible in the selected area (dashed black box).

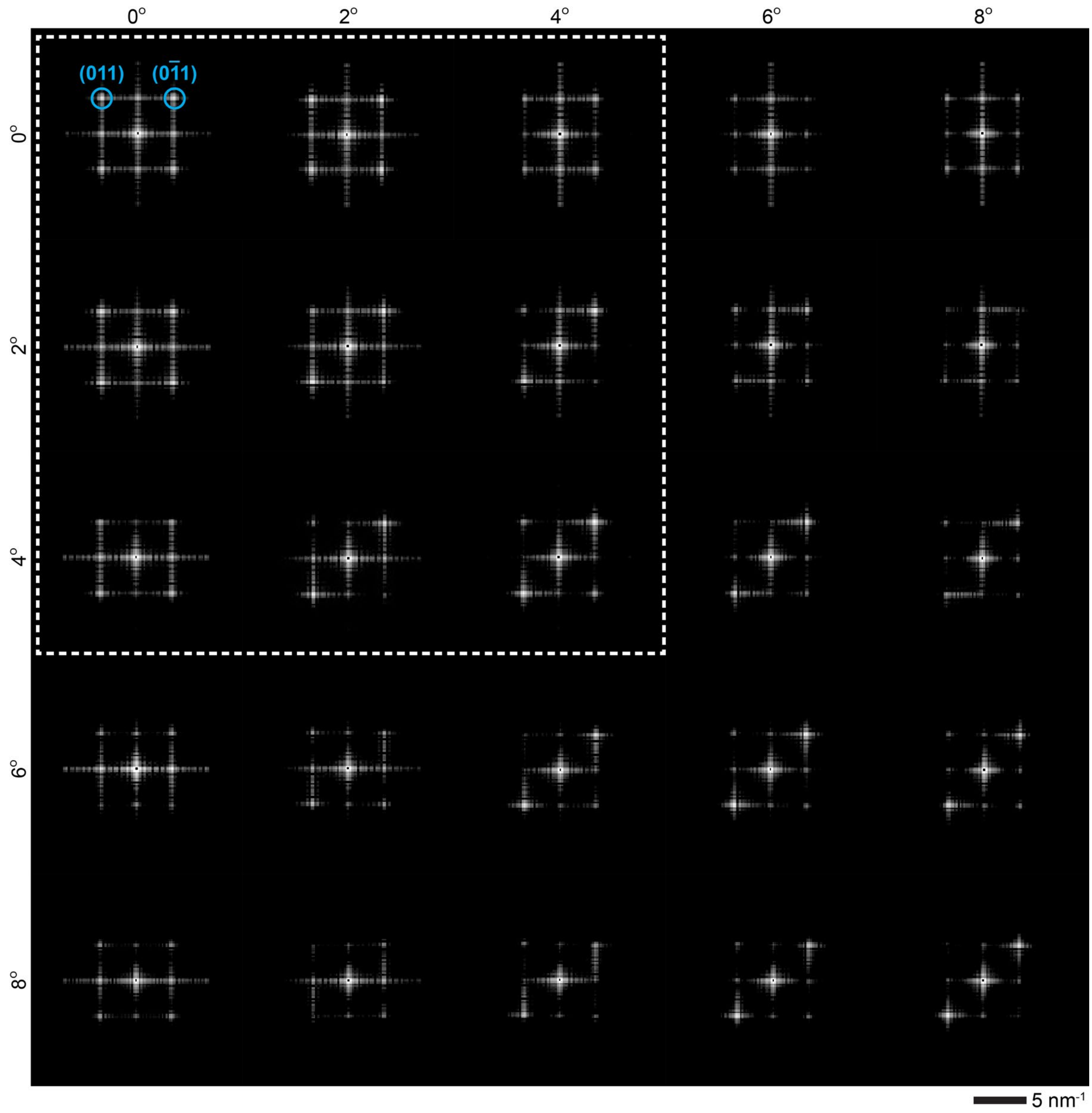

Supplementary Figure 19. FFT patterns corresponding to simulated images in Supplementary Figure 18. The (011) and $(0\bar{1}1)$ spots are visible in the selected area (dashed white box).

Supplementary Tables

Supplementary Table 1. Lattice-plane spacings of the fcc and B2 phases of PdCu alloy.

**fcc phase**		**B2 phase** (supplementary ref. [2])	
hkl	d_{hkl} (Å)	hkl	d_{hkl} (Å)
111	2.2	100	3.0
200	1.9	110	2.1
220	1.3	111	1.7
311	1.2	200	1.5
222	1.1	210	1.3
400	0.9	211	1.2

Supplementary Table 2. DFT-calculated relative energies for fcc and B2 phases of PdCu alloy. Here, the energy of the bulk B2 phase is set as the reference energy.

**fcc phase** (meV/atom)		**B2 phase** (meV/atom)	
bulk	36	bulk	0
{111}	288	{100}	233
{200}	381	{110}	224
{220}	528	{111}	271
		{211}	224

Supplementary Notes

1. Phase diagram of PdCu alloy

Supplementary Figure 2 shows the phase diagram of PdCu alloy system adapted from supplementary ref. 1. At the atomic compositions of our NPs (46% (at.) Pd and 54% (at.) Cu), the high-temperature (≥570 °C) stable phase is a disordered fcc phase, and the low-temperature (≤505 °C) stable phase is an ordered bcc, *i.e.*, B2 phase. At low temperatures used for our *in situ* heating studies (*e.g.*, 350−500 °C as indicated by a dashed red rectangle in Supplementary Figure 2), the as-synthesized PdCu NPs with a metastable fcc phase can transform into a thermodynamically stable B2 phase.

The lattice-plane spacings for fcc and B2 phases of PdCu alloys are listed in Supplementary Table 1. The fcc lattice-plane spacings were estimated by summing the corresponding Pd and Cu lattice-plane spacings (JCPDS no. 65-2867 for standard Pd and JCPDS no. 65-9026 for standard Cu) proportionally (*i.e.*, 46% (at.) Pd and 54% (at.) Cu) because according to the Vegard's law[3] the unit cell parameters should vary linearly with the composition of a uniform solid solution. The B2 lattice plane spacings are taken from supplementary ref. [2].

2. *In situ* phase transition of PdCu NPs viewed from different directions

Supplementary Figure 3 shows the fcc-to-B2 transition of a PdCu alloy NP viewed from $[110]_{\mathrm{fcc}}$ and $[211]_{\mathrm{B2}}$ zone axes. Similar to the case shown in Figure 1, the B2 phase nucleates at the surface of the NP (near the top left corner of the image) (Supplementary Figure 3b−c: t - t_0 = 11−65 s) and propagates inward into the NP until it fully transforms into a B2 NP (Supplementary Figure 3b−c: t - t_0 = 91−145 s). We also imaged a similar process from $[110]_{\mathrm{fcc}}$ and $[100]_{\mathrm{B2}}$ zone axes, and confirmed that the B2 phase propagates into the fcc phase in a similar manner (Supplementary Figure 4).

Supplementary Figure 5 shows the interface between fcc and B2 phases during the phase transition similar to that described in Figure 2 but viewed from $[110]_{\mathrm{fcc}}$ and $[100]_{\mathrm{B2}}$ zone axes instead of $[100]_{\mathrm{fcc}}$ and $[110]_{\mathrm{B2}}$. Here, the $(1\bar{1}\bar{1})_{\mathrm{fcc}}$ and $(0\bar{1}1)_{\mathrm{B2}}$ spots shown in the FFT pattern from the dashed white box region (Supplementary Figure 5a−b: t - t_0 = 6 s) overlap because the direction and fringe spacing of the $(1\bar{1}\bar{1})_{\mathrm{fcc}}$ planes ($d_{(1\bar{1}\bar{1})_{\mathrm{fcc}}}$ = 2.2 Å) remain almost unchanged when the planes evolve into the $(0\bar{1}1)_{\mathrm{B2}}$ planes ($d_{(0\bar{1}1)_{\mathrm{B2}}}$ = 2.1 Å). Meanwhile, the $(1\bar{1}1)_{\mathrm{fcc}}$ spot is connected to the $(011)_{\mathrm{B2}}$ spot by a "trace", and the angle between these sets of planes is 20° (Supplementary Figure 5a−b: t - t_0 = 6 s). As we explained in Figure 2, the "traces" in FFT patterns arise from the finite-width interface between the two phases. Hence, we can identify the fcc, interface, and B2 regions from the inverse FFT images by selecting the associated peaks (Supplementary Figure 5d). Initially, the fcc and B2 phases were at the right and left sides of the selected region, respectively. The interface appeared almost over the entire region in the projected TEM image (Supplementary Figure 5d: t - t_0 = 6 s), implying that the incident electron beam was more or less perpendicular to the interface plane. As the

phase transition continued, the fcc phase and the interface regions shrunk while the B2 phase expanded (Supplementary Figure 5d: t - t_0 = 50−55 s).

Supplementary Figure 6 shows the atomic configuration of a body-centered tetragonal (bct) unit cell constructed from two fcc unit cells (Supplementary Figure 6a) and projected views of both the bct and B2 unit cells from $[110]_{\mathrm{fcc}}$ and $[100]_{\mathrm{B2}}$ zone axes (Supplementary Figure 6b), supplementing the schematic illustrations shown in Figure 3. The bct unit cell is constructed to establish a direct one-to-one correspondence with the B2 unit cell. This relationship is known as Bain correspondence,[4] and it is often used to describe the displacive phase transitions. As established in an earlier study, while the phase transition in PdCu alloy has elements of diffusive transformation (*i.e.*, interlattice atomic diffusion of Pd and Cu), it also exhibits characteristics of displacive deformation (*i.e.*, collective displacement of lattice points),[5] justifying the use of Bain correspondence to describe the phase transition.[6-8]

In the projection image, the orientation of $(0\bar{1}1)_{\mathrm{B2}}$ planes are the same as that of $(\bar{1}1\bar{1})_{\mathrm{fcc}}$ planes, however, the $(011)_{\mathrm{B2}}$ planes are rotated by roughly 20° with respect to the $(\bar{1}11)_{\mathrm{fcc}}$ planes (Supplementary Figure 6b). The lattice plane spacings of these planes are $d_{(\bar{1}1\bar{1})_{\mathrm{fcc}}} = d_{(\bar{1}11)_{\mathrm{fcc}}} = 2.2$ Å *vs.* $d_{(0\bar{1}1)_{\mathrm{B2}}} = d_{(011)_{\mathrm{B2}}} = 2.1$ Å. Finally, the TEM images shown in Supplementary Figures 4−5 are consistent with the features from the projected views of the schematic illustrations in Supplementary Figure 6b.

3. Quantitative analysis of the fcc−B2 interface

To better visualize how the lattice planes shown in Figures 2c and 5b bend in the interface region of the NPs, we first identified and manually labeled the position of each lattice plane ($(1\bar{1}0)_{\mathrm{B2}}$−$(002)_{\mathrm{fcc}}$ in Figure 2c and $(\bar{1}01)_{\mathrm{B2}}$−$(002)_{\mathrm{fcc}}$ in Figure 5b). Next, we segmented the atomic columns inside each lattice plane by thresholding and assigned the center position of each column as its position. Finally, from the atomic column positions, we calculated the slopes between every two adjacent columns on the lattice plane, and these slopes represent the bending angles of the lattice plane. The bending angles were analyzed using our image processing algorithms written in Python 3.7[9] utilizing the libraries of OpenCV,[10] NumPy,[11] and SciPy.[9]

The angle between $(002)_{\mathrm{fcc}}$ and $(1\bar{1}0)_{\mathrm{B2}}$ planes is approx. 8° (Supplementary Figure 7c), and these planes are connected via an interface region in which the connecting planes bend gradually to link the planes of both phases (Supplementary Figure 7a−b). Most notably, as the B2 phase grows into the fcc phase, these phases remain separated by a 9-Å-wide coherent interface (Supplementary Figure 8).

Supplementary Figure 9 captures the evolution of the coherent interface at the onset of the nucleation of a B2 phase in the fcc NP. At the initial stage of the nucleation, $(002)_{\mathrm{fcc}}$ planes start to bend, forming an interface region that gradually propagates into the NP (Supplementary Figure 9c: t - t_0 = 12−73 s). Once the planes in this newly formed interface bend by 5°, the new B2 phase nucleates on the surface of the NP (Supplementary Figure 9c: t - t_0 = 73−167 s). The interface between fcc and the newly

nucleated B2 phase is coherent and remains so until the fcc NP transforms into a B2 NP (Supplementary Figure 9b).

4. Phase transition at different temperatures

To test the effect of the temperature on the fcc-to-B2 phase transition, we tracked the evolution of PdCu alloy NPs at two lower temperatures: 350 and 300 °C (Supplementary Figure 10). At 350 °C, as expected, the B2 phase continuously propagates across the NP (Supplementary Figure 10a: $t - t_0 = 0{-}300$ s). However, at 300 °C, the NP does not transform, and the B2 phase remains trapped within the same timescale (Supplementary Figure 10b: $t - t_0 = 0{-}360$ s). Our *in situ* results are in line with XRD measurements obtained from the ensemble of similar PdCu alloy NPs which finds that the fcc-to-B2 phase transition occurs at above 350 °C.[12]

5. Orientation relationship (OR) between fcc and B2 phases

To assess the impact of an electron beam used for *in situ* TEM imaging on the observed phase transition dynamics of the NPs, we first heated the as-synthesized NPs at 350 °C for 15 min inside the TEM with the beam blanked and then cooled the specimen down to an ambient temperature before imaging.

In Supplementary Figure 11a, the NP viewed from the $[110]_{\mathrm{fcc}}$ and $[100]_{\mathrm{B2}}$ zone axes displays the same crystallographic features as the NP in Supplementary Figure 5, meaning that $(\bar{1}11)_{\mathrm{fcc}}$ planes are parallel to $(01\bar{1})_{\mathrm{B2}}$ planes, and the $(1\bar{1}1)_{\mathrm{fcc}}$ and $(011)_{\mathrm{B2}}$ spots are roughly 20° apart and linked with the "trace", which corresponds to the fcc–B2 interface region. Also, comparing the NPs viewed from $[100]_{\mathrm{fcc}}$ and $[110]_{\mathrm{B2}}$ zone axes shown in Supplementary Figure 11b and Figure 2 reveal similar structures with the two spot pairs ($(002)_{\mathrm{fcc}}$–$(1\bar{1}0)_{\mathrm{B2}}$ spots and $(020)_{\mathrm{fcc}}$–$(002)_{\mathrm{B2}}$ spots) connected by "traces". Furthermore, the inverse FFT image in Supplementary Figure 11c from the selected region (dashed white box) in Supplementary Figure 11b shows that the fcc–B2 interface is a coherent interface aligned with the $(02\bar{2})_{\mathrm{fcc}}$ and $(1\bar{1}\bar{2})_{\mathrm{B2}}$ planes. To test whether the interface is along these directions at all times, we evaluated nine NPs viewed from $[100]_{\mathrm{fcc}}$ and $[110]_{\mathrm{B2}}$ zone axes (Supplementary Figure 11d) and found that seven out of nine times, interfaces are very close to the $\{220\}_{\mathrm{fcc}}$ planes, deviating by less than 10° from these planes.

To understand how crystal structure changes during the phase transition, we need to establish a 3D geometrical relationship between the two phases. Such a geometrical relationship, known as an orientation relationship (OR), can be specified by finding parallel planes between the two phases.[13] In Supplementary Figure 5, when viewing the fcc-to-B2 phase transition from the $[110]_{\mathrm{fcc}}$ and $[100]_{\mathrm{B2}}$ zone axes, the $(1\bar{1}\bar{1})_{\mathrm{fcc}}$ planes are parallel to the $(0\bar{1}1)_{\mathrm{B2}}$ planes. Furthermore, in Supplementary Figure 12, when viewing the phase transition from the $[111]_{\mathrm{fcc}}$ and $[110]_{\mathrm{B2}}$ zone axes, the $(\bar{2}02)_{\mathrm{fcc}}$ planes are parallel to the $(002)_{\mathrm{B2}}$ planes. Hence, we conclude that the OR between the fcc and B2 phases is Nishiyama–Wasserman OR,[13] in which $\{111\}_{\mathrm{fcc}}$ planes are parallel to $\{011\}_{\mathrm{B2}}$ planes, and $\{110\}_{\mathrm{fcc}}$ planes are parallel to $\{100\}_{\mathrm{B2}}$ planes.

6. Phase transition of large PdCu NPs

To check whether larger PdCu NPs behave similarly to the 8-nm NPs, we synthesized such NPs by using 16× less reducing agent (*i.e.*, 0.7 mg of ribose instead of 11.0 mg) while keeping everything else the same as described in the *Methods* section. The as-synthesized NPs were 13−37 nm in size and had an fcc structure (Supplementary Figure 13a). The EDX maps confirmed that their atomic compositions were 46% Pd and 54% Cu (Supplementary Figure 13b).

After heating these NPs at 350 °C for 30 min, we observed the nucleation and propagation of B2 phase into the fcc NPs (Supplementary Figure 14). Similar to the pathway of 8-nm NPs, here as well, B2 phase appears to nucleate from the edge of the fcc NPs, and then propagate across the NPs by forming a coherent interface (Supplementary Figure 15).

7. TEM simulations of the off-axis tolerance

We observed the phase transition dynamics from four different zone axes combinations of fcc and B2 phases: $[100]_{\mathrm{fcc}}$ and $[110]_{\mathrm{B2}}$ (Figures 1−2), $[110]_{\mathrm{fcc}}$ and $[111]_{\mathrm{B2}}$ (Figure 5), $[110]_{\mathrm{fcc}}$ and $[211]_{\mathrm{B2}}$ (Supplementary Figure 3), and $[110]_{\mathrm{fcc}}$ and $[100]_{\mathrm{B2}}$ (Supplementary Figures 4−5). From the OR between the two phases, as described in Supplementary Note 5 ($\{110\}_{\mathrm{fcc}}$ planes are parallel to $\{100\}_{\mathrm{B2}}$ planes, $\{111\}_{\mathrm{fcc}}$ planes are parallel to $\{011\}_{\mathrm{B2}}$ planes), the fcc and B2 zone axes are parallel only when viewed from $[110]_{\mathrm{fcc}}$ and $[100]_{\mathrm{B2}}$ zone axes (Supplementary Figures 4−5), whereas when viewed from other directions, the fcc and B2 zone axes appear apart by few degrees (approx. 7° in Figures 1−2 and approx. 6° in Figure 5). Nonetheless, a crystal structure is visible in TEM when imaged at slightly off-axis conditions.[14]

To test the off-axis tolerance in imaging the NPs, we simulated TEM and FFT images of fcc (Supplementary Figures 16−17) and B2 (Supplementary Figures 18−19) nanocrystals at different tilting angles (8°×8°). We built the atomic models of the NPs shown in Supplementary Figures 16 and S18 using CrystalMaker® 9.0.3 package[15] and then simulated the TEM and FFT images at different off-axis tilting conditions with QSTEM software.[16] The TEM parameters corresponding to the imaging conditions of the Thermofisher Titan TEM were used for our simulations. These parameters are as follows: accelerating voltage of 300 kV, C_s = 1.2 mm, convergence angle of 0.5 mrad, and Scherzer defocus.

These simulations in Supplementary Figures 16−19 reveal two crucial points. First, the lattices and the corresponding FFT spots are visible when the crystals are off-axis by few degrees in each axial direction (Supplementary Figures 16−19: dashed black and white boxes). Second, the position of each FFT spot remains unchanged, and only their intensities fade when tilting the crystal. These points justify our TEM results observed from $[100]_{\mathrm{fcc}}$ and $[110]_{\mathrm{B2}}$ zone axes (Figures 1−2) and $[110]_{\mathrm{fcc}}$ and $[111]_{\mathrm{B2}}$ zone axes (Figure 5).

Supplementary references

1. Huang, P., Menon, S. & de Fontaine, D. On the Cu-Pd phase diagram. *J. Phase Equil.* **12**, 3−5 (1991).
2. Gao, Q. *et al.* Shape‐controlled synthesis of monodisperse PdCu nanocubes and their electrocatalytic properties. *ChemSusChem* **6**, 1878−1882 (2013).
3. Jacob, K. T., Raj, S. & Rannesh, L. Vegard's law: a fundamental relation or an approximation? *Int. J. Mater. Res.* **98**, 776−779 (2007).
4. Porter, D. A., Easterling, K. E. & Sherif, M. Y. *Phase Transformations in Metals and Alloys* Ch. 6 (CRC Press, Boca Raton, 2009).
5. Subramanian, P. R. & Laughlin, D. E. Cu-Pd (copper-palladium). *J. Phase Equil.* **12**, 231−243 (1991).
6. Haghdadi, N. *et al.* New insights into the interface characteristics of a duplex stainless steel subjected to accelerated ferrite-to-austenite transformation. *J. Mater. Sci.* **55**, 5322−5339 (2020).
7. Beladi, H. *et al.* On the crystallographic characteristics of nanobainitic steel. *Acta Mater.* **127**, 426−437 (2017).
8. Toji, Y., Matsuda, H. & Raabe, D. Effect of Si on the acceleration of bainite transformation by pre-existing martensite. *Acta Mater.* **116**, 250−262 (2016).
9. Oliphant, T. E. Python for scientific computing. *Comput. Sci. Eng.* **9**, 10−20 (2007).
10. Bradski, G. The openCV library. *Dr. Dobb's J. Softw. Tools Prof. Program.* **25**, 120−123 (2000).
11. Van Der Walt, S., Colbert, S. C. & Varoquaux, G. The NumPy array: a structure for efficient numerical computation. *Comput. Sci. Eng.* **13**, 22−30 (2011).
12. Tong, W., Huang, B., Wang, P., Li, L., Shao, Q. & Huang, X. Crystal‐phase‐engineered PdCu electrocatalyst for enhanced ammonia synthesis. *Angew. Chem. Int. Ed.* **59**, 2649−2653 (2020).
13. Zhang, M.-X. & Kelly, P. M. Crystallographic features of phase transformations in solids. *Prog. Mater. Sci.* **54**, 1101−1170 (2009).
14. Williams, D. B. & Carter, C. B. *Transmission Electron Microscopy: A Textbook for Materials Science* Ch. 12 (Springer, Berlin, 1996).
15. CrystalMaker 9.0.3 (www.crystalmaker.com). CrystalMaker Software Ltd, Oxford, England.
16. QSTEM online (https://nanohub.org/resources/qstem).
