# Supplementary material for: Dynamics of the fcc-to-bcc phase transition in single-crystalline PdCu alloy nanoparticles
Source: Nat Commun. 2023 Jan 6;14:104. doi: 10.1038/s41467-022-35325-y (PMC9822937; doi:10.1038/s41467-022-35325-y)
Supplement: Supplementary file 3 — Description of Additional Supplementary Files [file 41467_2022_35325_MOESM3_ESM.pdf]

Supplementary Movie 1: Fcc-to-B2 phase transition of a PdCu NP at 500 °C viewed from $[100]_{fcc}$ and $[110]_{B2}$ zone axes, as shown in Figure 1.

Supplementary Movie 2: Fcc-to-B2 phase transition of a PdCu NP at 500 °C viewed from $[110]_{fcc}$ and $[211]_{B2}$ zone axes, as shown in Supplementary Figure 3.

Supplementary Movie 3: Fcc-to-B2 phase transition of a PdCu NP at 500 °C viewed from $[110]_{fcc}$ and $[100]_{B2}$ zone axes, as shown in Supplementary Figure 4.

Supplementary Movie 4: Movement of the fcc-B2 interface at 500 °C viewed from $[100]_{fcc}$ and $[110]_{B2}$ zone axes, as shown in Figure 2.

Supplementary Movie 5: Movement of the fcc-B2 interface at 500 °C viewed from $[110]_{fcc}$ and $[100]_{B2}$ zone axes, as shown in Supplementary Figure 5.

Supplementary Movie 6: Nucleation of B2 phase from fee phase during the heating ramp from 350 to 500 °C viewed from $[110]_{fcc}$ and $[111]_{B2}$ zone axes, as shown in Figure 5.
